# Making a Sustainable Diet Acceptable: An Emerging Programming Model With Applications to Schools and Nursing Homes Menus

**DOI:** 10.3389/fnut.2020.562833

**Published:** 2020-11-06

**Authors:** Luca Benvenuti, Alberto De Santis

**Affiliations:** Department of Computer, Control, and Management Engineering, Sapienza University of Rome, Rome, Italy

**Keywords:** diet acceptability, diet sustainability, nutrition, health, school lunch menu, nursing home menu

## Abstract

**Background:** Food consumption is one of the most important drivers of the relation between human well-being and Earth's ecosystems. The current production level is difficult to sustain without compromising environmental integrity or public health. This calls for a decisive change in food consumption patterns in order to improve nutrition quality while respecting biodiversity and ecosystems. This change will produce some effect only if it is also culturally acceptable, accessible, economically fair and affordable. The design of food plans is traditionally carried out using mathematical optimization models, such as linear programming. This method has proved to be successful in providing nutritionally adequate diets while minimizing their economic and environmental impact. Nevertheless, cultural habits as well as attractiveness and variety of meals is very difficult to deal with, and no fully satisfactory way to include these issues in linear programming has been found.

**Objective:** The aim of this paper is to move from traditional linear programming to a new programming methodology in order to cope also with acceptability in the design of meal plans.

**Method:** Binary integer linear programming is the new modeling paradigm. In the proposed model, meal plans consist of providing the sequence and composition of daily meals over a given period of time and each meal can be composed using dishes from a given set. Therefore, instead of defining just a level of consumption of food groups or food items, the proposed model provides a realistic menu. To cope with sustainability, the energy and nutritional content of each dish is calculated together with its price and environmental impact. Furthermore, acceptability can be explicitly taken into account in a very natural way, that is bounding the daily, weekly, or total repetitions of single dishes and of dishes in the same food groups.

**Results:** The paper reviews three successful studies with increasing complexity considering lunch plans for schools and full-board menus for nursing homes. The case studies show a great reduction of the environmental impact of the meal plans while ensuring an adequate nutritional intake, affordable prices and most importantly the plans are varied and culturally acceptable.

## 1. Introduction

By 2050, the global population is projected to reach 9 billions, which will nearly double the current global food demand. Achieving and sustaining production at that level are major challenges that must be met without compromising environmental integrity or public health (1). In this respect, agricultural technologies continue to make enormous advances in increasing crop production while safeguarding the environment. Nevertheless, this is not sufficient and it is necessary to explore other opportunities that may be viable in the long term in addressing sustainability of food production. Important opportunities are those of food waste reduction and a shift toward more sustainable lifestyles. In particular, a substantial shift in people eating patterns may help in improving nutrition quality through better balanced diets and may put pressure on resources for food production. In fact, nowadays, about one billion people still suffer from hunger, while even more people are overweight or obese and there is a high prevalence of micronutrient malnutrition (2). Moreover, there is strict relation between health of humans and that of ecosystems. For instance, consistent evidence indicates that a dietary pattern higher in plant-based foods (e.g., vegetables, fruits, legumes, seeds, nuts, whole grains) and lower in animal-based foods (especially red meat), as well as lower in total energy, is both healthier and associated with a lesser impact on the environment (3, 4). Further, food consumption patterns dictate the shape of the global food production system. For example, while substantial environmental impacts from food occur in the production phase (agriculture, food processing), households influence these impacts through their dietary choices and habits (5). Hence, food production, consumption together with nutrition define a complex system that calls for a more general definition of sustainability, addressing the environmental impact of the whole food supply chain, food and nutrition security. Sustainable diets are defined by the Food and Agriculture Organization of the United Nations (2) as “those diets with low environmental impacts which contribute to food and nutrition security and to healthy life for present and future generations. Sustainable diets are protective and respectful of biodiversity and ecosystems, culturally acceptable, accessible, economically fair and affordable; nutritionally adequate, safe and healthy; while optimizing natural and human resources.”

The design of a diet has then to integrate different dimensions of diet sustainability that may not be compatible with each other: health, environmental impact, cultural and socio-economic dimension.

The health dimension consists of promoting an adequate nutrition thus preventing chronic diseases. To this end nutritionists and various medical and governmental institutions provide evidence-based nutrition information and advice to help people in making healthy choices about food and beverages in their daily lives. This information mainly consists of dietary guidelines (6, 7) defining nutrient requirements, recommended nutrient intakes as well as recommended consumption level of some foods (8–10).

The environmental impact of food production relates to the level of greenhouse gas emissions (GHGE), the use of land and water resources, pollution, depletion of phosphorus, and the impact of chemical products, such as herbicides and pesticides (11).

Cultural habits, that is mealtime and meal composition, as well as food preference and preparation techniques, are greatly affected by traditions, beliefs and values shared by a community. Hence, they define the structure of each meal and the set of foods and dishes that are considered edible and acceptable (12). Moreover, when designing a meal plan, one has to consider also meals attractiveness and variability.

The socio–economic dimension is mainly related to food cost. In fact, price is the second most important factor impacting people's food choices after taste. Note that nutrition is considered only third in importance (13, 14).

These dimensions are generally conflicting. For instance, low cost diets corresponds to high energy density, whereas diets of higher nutrient density and nutritional quality have higher costs (15).

To comply with all these different competing dimensions, heuristic methods have been used. A typical approach is the substitution method consisting of replacing one or several foods with others in a given diet, to test the impact of the substitution on one dimension of the diet (16–23). A more sophisticated method consists of defining a score—measuring some dimension of the diet—and try to improve it by iterative substitution steps (24). These heuristic methods may well help testing effectiveness of some substitutions on a single aspect of a diet but are not suitable to efficiently deal with several competing dimensions. To achieve this goal, mathematical optimization models have proved to be successful. In more detail, these models are able to determine the optimal combination of foods with respect to some aspects of a diet while satisfying some predefined *constraints* (e.g., imposed nutrient recommendations, a total diet cost, an environmental target, etc.). Basically, they can provide food plans by minimizing an *objective function* given by either the environmental impact or the total expense for the diet (or a linear combination of them). The *food plan* consists of the level of consumption of selected food groups (such as fruit and vegetables, dairy, meat, fish, …) or food items (potatoes, carrots, beans, eggs, …) for person a day or a week. In literature, comprehensive studies of the use of linear programming (LP) to optimize diets taking into account their nutrition, economic and environmental impact dimensions are available (25, 26). In general, no practical *meal plans*—that is schedules of recipes—are provided when using LP. Indeed, only Macdiarmid (27) proposed a sample weekly meal plan in order to test whether the food items and the corresponding consumption levels in the optimal food plan could be heuristically combined into a realistic menu.

It is worth noting that besides sustainability dimension, acceptability is a key issue in a diet. Acceptability relies on palatability of foods and eating habits that, in turn, are determined by cultural factors, tradition and environmental conditions (28). In general, it may consist of selecting appropriate food groups (for example, some plants or animals may be considered edible or not, depending of cultural and religious habits), in serving a varied ensemble of recipes (pasta can be served with several different sauces and condiments), including traditional dishes (for example pizza or lasagna in Italy), and with a given weekly frequency. In the context of LP, there have been several attempts to include acceptability issues when designing a diet. This is usually accomplished in an indirect way either defining appropriate constraints on consumption of some food groups (29, 30), or introducing a suitable penalty score in the objective function (31, 32). Despite these attempts, no study has provided the ultimate solution to take into consideration acceptability issues (25) and the need of a new modeling paradigm is evident. In fact, while LP has proved to be effective in finding solutions to a variety of complex diet problems, it appears to be quite unsuitable to tackle acceptability since it does not provide a meal plan but just a food plan. Hence, it results very difficult, for example, to consider issues such as the frequency of recipes and/or food items in the plan in order to make it varied and attractive according to people's eating habits.

In this paper a new modeling paradigm to design sustainable diets is provided. The meal plan consists of providing the sequence and composition of daily meals over a given period of time. Each meal can be composed using dishes from a given set. Therefore, instead of defining just a level of consumption of food groups or food items, the proposed model provides a realistic menu. To this end, the composition of the meals, i.e., its structure and the set of recipes, is defined according to the cultural habits. Moreover, the energy and nutritional content of each dish is calculated together with its price and environmental impact so that the plan can be designed while complying with all the other dimensions of the diet. This is achieved by defining appropriate criteria for dishes selection according to the competing goals of a sustainable diet while ensuring meals attractiveness and variability. From a mathematical point of view, a binary variable is associated to each dish for every meal, and it denotes the presence or absence of the dish in the meal. Therefore, acceptability, attractiveness and variability of the diet can be explicitly addressed at the cost of a more complex optimization problem than LP, that is a 0–1 binary integer linear programming (BLP) problem.

This approach is a well-established and validated practice in engineering problems, such as, for example, industrial production planning, services scheduling, and frequency planning in telecommunication networks (33). In this context, the proposed methodology considers a menu as an optimal allocation of resources (dishes) over the period of the diet.

To show the effectiveness of the approach, the paper reports the results of three recent studies (34–36) regarding the design of lunch menus for schools and full-board menus for nursing homes. In these studies, the methodology is applied with increasing level of complexity of the acceptability dimension. The reported results show a great reduction of the environmental impact of the meal plans while ensuring an adequate nutritional intake, affordable prices and most importantly the plans are varied and culturally acceptable.

## 2. Methods

A menu, consists of providing the sequence and composition of daily meals over a given period of time. This can be done by selecting dishes from a given set of *N* recipes of fixed portion size^1^. The design of a menu can therefore be modeled as the assignment of dishes (resources) to given places in a time schedule (slots). The number of slots depends on:

- The kind of menu: it can be an half board menu, a full board menu or, in general, a menu with a number of *N*_*M*_ meals per day;
- The number of days: the service can be full week, as for example in hospitals, or workweek, as for company canteens. In general, the service is over a number *N*_*D*_ of days in a week;
- The number *N*_*W*_ of weeks.

A slot is then identified by a set of three indexes *m*, *d*, and *w*, denoting the meal *m* of the day *d* of the week *w* in the menu. A binary variable $x_{m,d,w}^{i}$ is associated to every dish and every slot and it assumes value 1 if the dish *i* is served in the slot *m*, *d*, *w*, and 0 otherwise. The index *m* takes values in a subset

$$\begin{array}{l} M\subseteq \{breakfast,mid-morning\;snack,lunch,\\ \;mid-afternoon\;snack,dinner\} \end{array}$$

while the index *d* takes values in a subset

$$\begin{array}{l} D\subseteq \left\{mon,tue,wed,thu,fri,sat,sun\right\} \end{array}$$

Finally, the index *w* takes values in a set *W* = {1, …, *N*_*W*_} for a menu of *N*_*W*_ ≥ 1 weeks. Hence, for example,

$$\begin{array}{l} x_{dinner,mon,2}^{i}=1 \end{array}$$

means that the dish *i* is served in the Monday dinner of the second week of the menu. Therefore, the design of a meal plan consists in assigning the values of the *N* × *N*_*M*_ × *N*_*D*_ × *N*_*W*_ variables $x=\left\{x_{m,d,w}^{i}\right\}$. This assignment must be performed integrating the different dimensions of diet sustainability, that is: health, environmental impact, cultural and socio-economic dimension. Moreover, meals attractiveness and variability must be ensured.

### 2.1. Health Dimension

Nutrition plays a crucial role in health promotion and chronic disease prevention. To this end nutritionists and various medical and governmental institutions provide nutrition information and advice on healthy choices about food and beverages. As a matter of fact, Dietary Reference Intakes (DRIs) and Dietary Reference Values (DRVs) (6–10) define the proportion of a person's total energy intake as well as the expected nutritional content, which should come from different components of food. These values, which may vary by age, weight, gender, level of physical activity …, may include:

- **EARs:** Estimated Average Requirements, an estimate of the average requirement of energy or a nutrient that satisfies the needs of 50% of the people;
- **RDAs, RNIs:** Recommended Dietary Allowances or Reference Nutrient Intakes, the daily dietary intake level of a nutrient considered sufficient to meet the requirements of 97.5% of healthy individuals;
- **AIs:** Adequate Intakes, where no RDAs have been established;
- **ULs:** Tolerable upper intake levels, the highest level of daily nutrient consumption that is considered to be safe for, and causes no side effects in 97.5% of healthy individuals;
- **LRNIs:** Lower Recommended Nutritional Intakes, the lowest level of daily nutrient consumption that is enough in 2.5% of healthy individuals;
- **AMDRs:** Acceptable Macronutrient Distribution Ranges, ranges of intakes specified as a percentage of total energy intake. They are used for sources of energy, such as fats and carbohydrates.

In order to comply with these recommendations some parameters *p*, such as energy and nutrients content (*lipid, sugar, fiber*, etc., …), are associated to each dish. For example, when considering lunch for primary school, that is for children 6–10 years old, the dish *pasta with tomato sauce*^2^ provides 171.46 kcal, 6.08 g of proteins, 24.32 g of carbohydrates, 6.17 g of fats, 4.02 g of sugars, 2.01 g of fibers, and 45.95 mg of sodium (35). The recommendations can then be formulated as lower and/or upper bounds (box constraints) on the values of these parameters for each meal or all the meals in a day. To this aim, denote by $q_{i}^{p}$ the value of parameter *p* of the dish *i*, so that the quantity of the parameter *p* in the meal of the slot *m*, *d*, *w* is

$$\begin{array}{l} Q_{m,d,w}^{p}\left(x\right)=\overset{N}{\underset{i=1}{\sum }}x_{m,d,w}^{i}\cdot q_{i}^{p} \end{array}$$

and therefore the daily quantity of the parameter *p* is

$$\begin{array}{l} Q_{d,w}^{p}\left(x\right)=\underset{m\in M}{\sum }Q_{m,d,w}^{p}\left(x\right)=\underset{m\in M}{\sum }\overset{N}{\underset{i=1}{\sum }}x_{m,d,w}^{i}\cdot q_{i}^{p} \end{array}$$

Note that both the quantities $Q_{m,d,w}^{p}\left(x\right)$ and $Q_{d,w}^{p}\left(x\right)$ are linear combination of the variables $x_{m,d,w}^{i}$. Hence, daily recommendations can be modeled by linear constraints as follows:

$$\begin{array}{l} L_{p}\leq Q_{d,w}^{p}\left(x\right)\leq U_{p} \end{array}$$

where *L*_*p*_ and *U*_*p*_ are the bounds defined by DRIs, DRVs, or AMDRs. For example, the AMDR for carbohydrates for both males and females aged 19−70 years, is 45−65% of total calories. Hence, for a diet of 2,000 kcal a day, one has^3^

$$\begin{array}{l} L_{carbs}=225g\leq Q_{d,w}^{carbs}\left(x\right)\leq U_{carbs}=325g \end{array}$$

Moreover, some further recommendations consist in limiting or avoiding the consumption of some food groups and increasing that of others. For example, public health authorities recommend consuming more plant-based foods, a limited amount of animal products, especially red meat, and avoiding eating processed meet as well as alcohol drinking. To take into account such a kind of recommendations, some food groups are defined (red meat, vegetables, dairy, etc., …) and each dish is assigned to the proper food group. For example, beef burger and veal cutlet recipes are part of the “red meat” food group and cancer prevention recommendations (38) limit their consumption to no more than about three portions per week, that is equivalent to about 350–500 grams of cooked weight. This recommendation can then be described considering the weekly rate of red meat consumption, i.e.,

$$\begin{array}{l} R_{w}^{red\;meat}\left(x\right)=\underset{d\in D}{\sum }\underset{m\in M}{\sum }\underset{\;i\in red\;meat}{\sum }x_{m,d,w}^{i} \end{array}$$

and constraining it as follows:

$$\begin{array}{l} R_{w}^{red\;meat}\left(x\right)\leq 3 \end{array}$$

Note that, also in this case, a linear constraint over the variables $x_{m,d,w}^{i}$ is obtained.

### 2.2. Cultural Dimension

There are many factors that determine what foods a person eats. In addition to personal preferences, there are cultural, social, religious, economic, environmental, and even political factors. As a matter of fact, a cultural group provides guidelines regarding acceptable foods, food combinations, and eating patterns. In particular:

- **Acceptable foods**. What is considered acceptable is mainly driven by cultural and religious factors. For example in the West, regardless religious believes, eating dogs is generally not considered acceptable, while this animal is eaten without any particular problem in Korea, Vietnam, and China. Religious dictates, however, tend to have broader and stricter prohibitions (39). For example, the pork meat is forbidden in Islam and that of rabbit in Judaism.
- **Food combinations and eating patterns**. A meal is usually defined as the consumption of two or more foods in a structured setting at a set time. A common eating pattern is three main meals (breakfast, lunch, and dinner) per day, with snacks between meals. The composition of each meal varies across cultures, but generally include one or more courses, which usually correspond to one dish. In the Mediterranean area, for example, typical lunches and dinners are composed of a first course, a second course and a side dish. Moreover, also food combinations of dishes greatly depend on gastronomic tradition.

Hence, to comply with cultural habits it is firstly necessary to define a proper set of recipes from which to select the dishes of the menu. This set must respect dietary habits and choices and contain dishes from the local cuisine and traditional foods. Then, the meal plan has to respect the habitual structure of the meals. To this end, each dish is assigned to the proper course, i.e., first course, second course, side dish, etc., …, so that the meal structure can be guaranteed by appropriate constraints over the variables $x_{m,d,w}^{i}$. For example, when considering a typical lunch in the Mediterranean area, its structure is guaranteed by:

$$\begin{array}{l} R_{lunch,d,w}^{first}\left(x\right)=\underset{i\in first}{\sum }x_{lunch,d,w}^{i}=1, \end{array}$$

$$\begin{array}{l} R_{lunch,d,w}^{second}\left(x\right)=\underset{i\in second}{\sum }x_{lunch,d,w}^{i}=1, \end{array}$$

$$\begin{array}{l} R_{lunch,d,w}^{side}\left(x\right)=\underset{i\in side}{\sum }x_{lunch,d,w}^{i}=1 \end{array}$$

On the other hand, attractiveness and variability of the meal plan can be pursued by fixing the minimum and maximum number of times that dishes of the same food group^4^ can be served in a day, a week or in the whole menu. For example, in a varied menu, a pasta dish may be served at most once in a day and not for every day in a week, say at most four times in a week. This requirement can then be expressed as box constraints over the daily and weekly rates of pasta dishes as follows:

$$\begin{array}{lll} R_{d,w}^{pasta}\left(x\right) & = & \underset{m\in M}{\sum }\underset{\;i\in pasta}{\sum }x_{m,d,w}^{i}\leq 1,\\ R_{w}^{pasta}\left(x\right) & = & \underset{d\in D}{\sum }\underset{m\in M}{\sum }\underset{\;i\in pasta}{\sum }x_{m,d,w}^{i}\leq 4 \end{array}$$

Moreover, attractiveness and variability can be further ensured by constraining even the single dish rate in a week or in the whole menu. For example, the following constraints:

$$\begin{array}{lll} R_{w}^{i}\left(x\right) & = & \underset{d\in D}{\sum }\underset{m\in M}{\sum }x_{m,d,w}^{i}\leq 2,\\ R^{i}\left(x\right) & = & \underset{w\in W}{\sum }\underset{d\in D}{\sum }\underset{m\in M}{\sum }x_{m,d,w}^{i}\leq 5 \end{array}$$

impose that the dish *i* is served at most two times in a week and no more than five times in the entire menu. Again, linear constraints over the variables $x_{m,d,w}^{i}$ are obtained.

### 2.3. Environmental Impact Dimension

The impact of food production, that is livestock, fisheries and agriculture, on the environment is very significant. The following points give a general idea of the extent of this impact (40):

- Food accounts for over a quarter (26%) of global greenhouse gas emissions (41);
- Half of the world's habitable (ice- and desert-free) land is used for agriculture;
- 70% of global freshwater withdrawals are used for agriculture (42);
- 78% of global ocean and freshwater eutrophication (the pollution of waterways with nutrient-rich pollutants) is caused by agriculture (41);
- 94% of mammal biomass (excluding humans) is livestock. This means livestock outweigh wild mammals by a factor of 15-to-1 (43).

Food production is therefore strategical in trying to tackle climate change, reducing water stress, pollution, restoring lands back to forests or grasslands, and protecting the world's wildlife. The impact of food production on the environment can be characterized by some standard consumption-based indicators, such as:

- **Land footprint:** the land used to produce one kilogram of food product. For example, the land use of meat from beef cattle, in industrial systems, is between 15 and 29 *m*^2^*y*/*kg* of which grassland 2−26 *m*^2^*y*/*kg*. On the contrary, the land use for eggs is between 4 and 7 *m*^2^*y*/*kg* and no grassland is required (44).
- **Carbon footprint:** the greenhouse gas emitted to produce one kilogram of food product. It is expressed as carbon dioxide equivalent and takes into account all the primary greenhouse gases, i.e., carbon dioxide *CO*_2_ methane *CH*_4_ and nitrous oxide *N*_2_*O*;
- **Water footprint:** the freshwater withdrawals required to produce one kilogram of food product. For example, the water footprint of meat from beef cattle is 15,400 *m*^3^/*ton* (as a global average) and is much larger than that of meat from chicken, which is 4,300 *m*^3^/*ton* (45).
- **Eutrophication:** the eutrophying emissions due to the production of one kilogram of food product. Eutrophication is the pollution of water bodies and ecosystems with excess nutrients and the leading emission is the runoff of nitrogen and other nutrients from agricultural production systems.
- **Ecological footprint:** it measures the ecological assets that a given population requires to produce the natural resources it consumes (including plant-based food and fiber products, livestock and fish products, timber and other forest products, space for urban infrastructure) and to absorb its waste, especially carbon emissions. It tracks the use of six categories of productive surface areas: cropland, grazing land, fishing grounds, built-up land, forest area, and carbon demand on land (46).

The environmental impact of a menu can then be evaluated from the above indicators considering the production of the foods composing the dishes in the menu. For example, the carbon and water footprints of the dish *pasta with tomato sauce* can be computed summing up the GHG emitted or the water consumed to produce the ingredients, thus obtaining 260.73 g of *CO*_2,*eq*_ and 250,00 L of water (35). In order to take into account the environmental impact of the diet plan, footprint indicators are computed and associated to each dish. For example, denoting by $q_{i}^{CF}$ the carbon footprint of the *i*-th dish, the carbon footprint of the menu is

$$\begin{array}{l} Q^{CF}\left(x\right)=\underset{w\in W}{\sum }\underset{d\in D}{\sum }\underset{m\in M}{\sum }\overset{N}{\underset{i=1}{\sum }}x_{m,d,w}^{i}\cdot q_{i}^{CF} \end{array}$$

and can be constrained by an appropriate upper bound. Even in this case, the constraint is linear with respect to the variables $x_{m,d,w}^{i}$.

### 2.4. Economic Dimension

It is well-known that food costs influence diet quality. As a matter of fact, food and nutrition played a key part in social inequalities in health, with poor health resulting from buying foods richer in energy (high in fat and sugar) to satisfy hunger, which are much cheaper per unit of energy than foods rich in protective nutrients (like fruits and vegetables) (47). Healthy diets with low environmental impacts that are culturally acceptable have an actual impact on natural and human resources only if they are adopted by the most of the population. To this aim, they must be also economically affordable. The cost of a diet is then a key issue in determining the effectiveness of any sustainable policy for food production and consumption. The cost of food depends on different factors, such as farm production, processing, manufacturing, wholesaling, distribution, and retail. All these factors, in turn, vary among producers, geography, production volume, and technology and can fluctuate due to the seasons. Therefore, in order to further characterize dishes by their costs, food prices should be referred to a precise location and averaged over different retailers and brands and over a given period of time. Hence, the cost of a dish can be computed starting from its ingredient; for example the cost of *pasta with tomatoes sauce* is obtained summing up the cost of the ingredients, thus obtaining an average price equal to 0.42 euros (computed in 2019 at Rome, Italy).

In order to take into account the economic dimension of the diet plan, prices are computed and associated to each dish. For example, denoting by $q_{i}^{price}$ the price of the *i*-th dish, the menu costs

$$\begin{array}{l} Q^{price}\left(x\right)=\underset{w\in W}{\sum }\underset{d\in D}{\sum }\underset{m\in M}{\sum }\overset{N}{\underset{i=1}{\sum }}x_{m,d,w}^{i}\cdot q_{i}^{price} \end{array}$$

and can be constrained by an appropriate upper bound. Again this constraint is a linear function of the variables $x_{m,d,w}^{i}$.

### 2.5. Selection Criteria

As previously discussed, to comply with sustainability, some parameters are associated to each dish:

- **Nutritional parameters:** they correspond to energy content and macronutrient (carbohydrates, proteins, fats) and micronutrient (minerals, vitamins, …) contents of the dish;
- **Environmental impact parameters:** they correspond to the land, carbon, water, eutrophication and ecological footprints of the dish;
- **Economic parameter:** it is the cost of the dish.

Moreover, cultural and health issues, as well as attractiveness and variability of the plan, require every dish to be characterized by some of the following groups:

- **Food groups:** they may be very general (cereals, dairy, vegetables, …) and/or more detailed (pasta, rice, bread, …, yogurt, cheese, milk, …);
- **Use groups**: they correspond to the possible position or role of dishes in the meal (first course, second course, side dish, …).

Nutritional and healthy guidelines, as well as environmental impact and plan cost, can then be implemented by constraining within a minimum and a maximum value the quantities

$$\begin{array}{l} Q_{m,d,w}^{p}\left(x\right),\;Q_{d,w}^{p}\left(x\right),\;Q_{w}^{p}\left(x\right),\;Q^{p}\left(x\right) \end{array}$$

of parameter *p* in a meal, in a day, in a week or in the entire menu.

Health recommendations, as well as cultural habits and attractiveness and variability can be implemented by constraining the rates

$$\begin{array}{l} R_{m,d,w}^{g}\left(x\right),\;R_{d,w}^{g}\left(x\right),\;R_{w}^{g}\left(x\right),\;R^{g}\left(x\right) \end{array}$$

and

$$\begin{array}{l} R_{m,d,w}^{i}\left(x\right),\;R_{d,w}^{i}\left(x\right),\;R_{w}^{i}\left(x\right),\;R^{i}\left(x\right) \end{array}$$

that are the number of times that dishes in the group *g*, and the *i*th dish, respectively, are served in a meal, in a day, in a week or in the entire menu.

Once all the constraints characterizing sustainability are fixed, all the plans satisfying them are healthy and attractive, culturally acceptable, environmental friendly and affordable. These plans differ either for the served dishes or for the order in which they are served. Moreover, they may have different energy and nutrient contents, different values for the environmental indicators, and different costs. It is therefore possible to select a precise plan among all these sustainable plans, according to some criteria. For example, it may be possible to choose the plan with minimum cost, the one with minimum GHG emissions, that with maximum content of iron, etc., …. In general, the criteria correspond to select the “best” sustainable plan with respect to some goal. The goal is usually chosen among the quantities *Q*^*p*^(*x*) for one or more parameter *p* (i.e., the total amount of the parameter *p* in the whole plan) and therefore it is a linear function of the variables $x_{m,d,w}^{i}$. From a mathematical point of view, this is a BLP problem, that is an optimization problem over binary variables with a linear objective function and linear constraints.

The problem of selecting the “best” sustainable plan with respect to some goal defined over some quantities *Q*^*p*^(*x*) is usually denoted as

$$\begin{array}{l} x^{⋆}:f\left(x^{⋆}\right)=\underset{x\in F}{\text{min }}f\left(x\right) \end{array}$$

where:

- *x* denotes a possible plan, that is a set of values of the variables $x_{m,d,w}^{i}$ assigning dishes to all available slots in the plan;
- F is the so called *feasible set* and represents the set of values of *x* satisfying the constraints, that is all the sustainable plans;
- *f*(*x*) is the so called *objective function*, and it represents the goal of the selection criterion. In the context of plan design, it is usually a linear combination of the quantities *Q*^*p*^(*x*) for some parameters *p*;
- *x*^⋆^ is then the “best sustainable plan”, i.e., the plan that is sustainable and minimizes the goal *f*(*x*).

For example, when the goal is that of minimizing the cost of the plan, then *f*(*x*) = *Q*^*price*^(*x*). On the other hand, if the goal is that of maximizing the content of iron in the whole plan, then *f*(*x*) = −*Q*^*iron*^(*x*). Finally, the goal can be a linear combination of two or more quantities *Q*^*p*^(*x*): for example, minimizing the environmental impact of the plan by means of the carbon and water footprints can be done by considering the following objective function

$$\begin{array}{l} f\left(x\right)=c_{1}\cdot Q^{CF}\left(x\right)+c_{2}\cdot Q^{WF}\left(x\right) \end{array}$$

where *c*_1_ and *c*_2_ determine a trade off between the two footprints. In this case, the best sustainable plan *x*^⋆^ minimizes none of the two footprints but just a given blend of them. A different plan *x*^⋆^ is obtained when changing the coefficients *c*_1_ and *c*_2_ thus shifting the relative importance of one footprint with respect to the other in the selection criterion. This can be done in a more systematic way by considering a multi–objective optimization problem, that is an optimization problem with more than one goal. In this case, the problem is usually denoted as

$$\begin{array}{l} \underset{x\in F}{\text{min}}\left(f_{1}\left(x\right),f_{2}\left(x\right),\ldots ,f_{k}\left(x\right)\right) \end{array}$$

where the objective functions *f*_*i*_(*x*) are conflicting, that is no single “best sustainable plan” *x*^⋆^ exists that simultaneously optimizes each objective. In this case, when two different plans *x*_1_ and *x*_2_ are compared, *x*_1_ is said to *dominate* *x*_2_ if

$$\begin{array}{l} f_{i}\left(x_{1}\right)\leq f_{i}\left(x_{2}\right)\text{ for all indexes }i=1,\ldots ,k \end{array}$$

$$\begin{array}{l} f_{j}\left(x_{1}\right)<f_{j}\left(x_{2}\right)\text{ for at least one index }j=1,\ldots ,k \end{array}$$

In words, all the goals corresponding to the plan *x*_1_ are not worse than those of plan *x*_2_ and at least one is strictly better.

**Figure 1:**
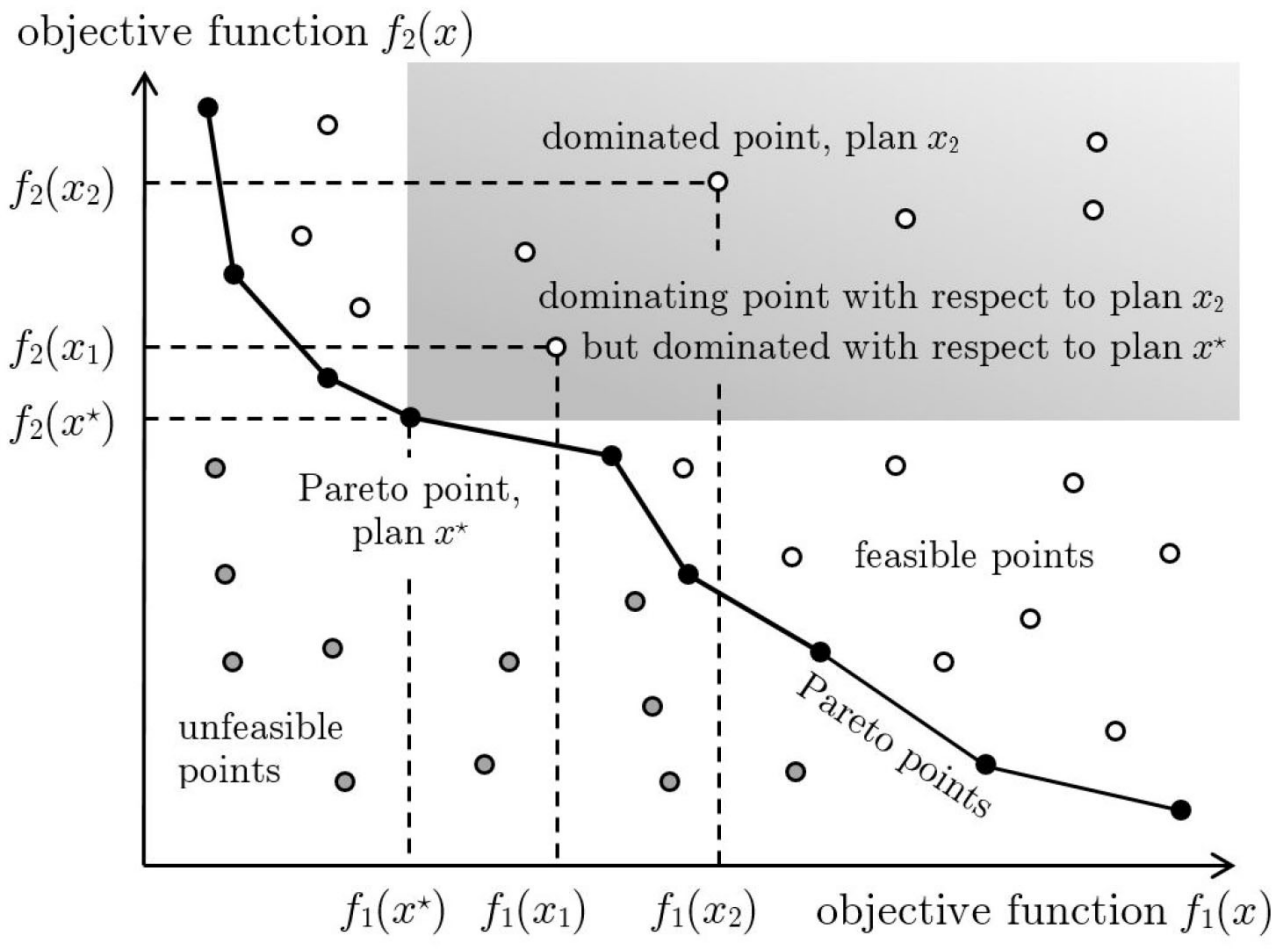
The white circles represent feasible points, that is those corresponding to sustainable plans. The gray circles represent unfeasible points, that is those corresponding to plans that are not sustainable since at least one of the constraints is violated. The black circles represent Pareto points, that is sustainable plans non-dominated by any other sustainable plan. For example, the sustainable plan *x*_2_ is dominated by the sustainable plan *x*_1_ which, in turn, is dominated by the sustainable plan *x*^⋆^. The latter cannot be dominated by any other sustainable plan and hence correspond to a Pareto point. All the sustainable plans dominated by *x*^⋆^ fall within the gray area.

This is graphically illustrated in Figure 1 in which the case of a multi–objective optimization problem with two objective functions is considered. In particular, two sustainable plans *x*_1_ and *x*_2_ are considered and the corresponding points in the plane of the objective functions are depicted. It is then easy to verify that the plan *x*_1_ dominates the plan *x*_2_ since *f*_1_(*x*_1_) < *f*_1_(*x*_2_) and *f*_2_(*x*_1_) < *f*_2_(*x*_2_). In a multi–objective optimization problem attention must be given to the *Pareto optimal solutions*, that is sustainable plans that cannot be improved in any of the goals without deteriorating at least one of the others. The set of the Pareto optimal solutions is called the *Pareto optimal set* and collects all the sustainable plans that are not dominated by any other sustainable plan. This set is very important since the trade–off between objectives can be made within this set, rather than considering the whole range of sustainable plans.

The Pareto optimal set is usually computed scalarizing the problem, that is by combining its multiple objectives into one single-objective scalar function. In other words, the problem is scalarized by defining an appropriate sequence of single–objective optimization problems whose optimal solutions are the Pareto optimal solutions of the multi–objective optimization problem^5^ (48). Several scalarizing methods are known. Among these, the simpler are:

- The **weighted–sum method** that consists of minimizing a positively weighted convex sum of the objectives (49). For example, in the case of two objective functions, the Pareto optimal set is obtained by choosing *c*_1_ = α, *c*_2_ = 1 − α and solving the sequence of scalar optimization problems
  $$\begin{array}{l} \underset{x\in F}{\text{min}}\left(\alpha \cdot f_{1}\left(x\right)+\left(1-\alpha \right)\cdot f_{2}\left(x\right)\right) \end{array}$$
  for 0 ≤ α ≤ 1;
- The ϵ–**constraint method** that consists of minimizing one objective when applying upper bounds to all the others (50). In this case, the Pareto optimal set is obtained by solving the sequence of scalar optimization problems
  $$\begin{array}{l} \underset{x\in F}{\text{min }}f_{1}\left(x\right) \end{array}$$
  such that *f*_2_(*x*) ≤ ϵ for ϵ_*min*_ ≤ ϵ ≤ ϵ_*max*_ where
  $$\begin{array}{l} \epsilon_{min}=\underset{x\in F}{\text{min }}f_{2}\left(x\right),\;\epsilon_{max}=\underset{x\in F}{\text{max }}f_{2}\left(x\right) \end{array}$$

### 2.6. Source of Data

The set up of the BLP problem previously described requires:

1. The definition of the meal plan characteristics, that is the kind of menu considered (full–board, half–board, …);
2. The definition of the related *use* and *food* groups;
3. The selection of a most varied possible set of dishes of fixed portion size, i.e., as rich as possible in the recipes composing each one of the defined use and food groups;
4. The selection of the nutritional, environmental impact and economic parameters of interest;
5. Data about the parameter values of every dish;
6. Data about the constraints ensuring sustainability of the plan;
7. The definition of the selection criterion, that is the objective functions of the problem.

Further information can be given about some of the points above. In the relevant literature about this modeling technique, the set of recipes used to compose the menu was either provided by catering companies (34) or by local authorities^6^ (35) or retrieved from a sample of available menus (36). The data about the nutritional parameter values of every dish have been computed from its ingredient using the food composition databases of the national agencies for food and drug. In particular, the Spanish database (51) was used in Ribal et al. (34), the Italian database (52) in Benvenuti et al. (35), and the French database (53) in Benvenuti et al. (36).

The data about the environmental impact parameter values have been assessed either from the literature dealing with life cycle assessment and studies into food products (34), or from available databases [the World Wide Fund for Nature database (54)^7^ for CF and WF was used in Benvenuti et al. (35), the Carbon Scope Data LCI database (57) was used in Benvenuti et al. (36)]. The economic impact parameter values of every dish have been computed from the market prices of its ingredients; these prices were made available by local wholesalers or collecting them from a sample of local stores. Finally, the data about the constraints were retrieved from the Spanish literature (58) in Ribal et al. (34) and from national institutions (10) in Benvenuti et al. (35, 36).

As for the implementation and numerical solution of the BLP problem, several tools are available. In particular, LINGO© has been used in Ribal et al. (34) and AMPL© in Benvenuti et al. (35, 36). Both of them integrate a proprietary language for expressing optimization models and provide several built-in solvers for linear, non-linear, and integer programming.

## 3. Results

In this section three case studies are illustrated. These cases have been recently presented in references (34–36) and make use of the optimization model proposed in this paper with different levels of difficulties. The first two cases consider the design of school lunch plans for primary schools over a period of 4 weeks (20 days). The last case is more complex since a 2 weeks full–board meal plan for nursing homes is presented. The description of the case studies focuses on the model structure and on the level of complexity of the acceptability constraints. The interested reader is referred to the corresponding reference for further details and for the values of the model parameters that are of interest of nutritionists and dietitians (list of recipes considered along with their energy and nutrients content, footprints and price, lower and upper bounds of the constraints).

### 3.1. Case 1: A 20-Days School Lunch Plan

The case study (34) consists of finding optimal lunch plans for school children in Spain for a 20-days (5 days a week for 4 weeks) planning period. Hence, *N*_*M*_ = 1, *N*_*D*_ = 5 and *N*_*W*_ = 4. A total set of *N* = 47 dishes is considered and they are chosen with slightly modification among the typical dishes served by a school catering company. Therefore, the number of binary variables is equal to *N* × *N*_*M*_ × *N*_*D*_ × *N*_*W*_ = 940. The following parameters are associated to each dish:

- 15 **nutritional parameters:** the caloric content together with 14 among macronutrients and micronutrients (protein, fat, carbohydrate, fatty acids, cholesterol, fiber, sodium, calcium, iron, potassium, magnesium, vitamin A, vitamin C, and vitamin E) contents of the dish are calculated from its ingredients using the database of the Spanish Agency for Food Safety and Nutrition (51);
- 1 **environmental impact parameter:** the carbon footprint of the dish is calculated from the ingredients on the basis of literature on life cycle assessment and carbon footprint studies on food products;
- 1 **economic parameter:** the cost of the dish is calculated from the market prices of the raw foods. The source for food prices is one of the most important wholesalers in Spain. Only the price of raw materials is taken into account and neither labor costs nor other direct costs are computed.

Moreover, in order to take into account the cultural habits and to guarantee attractiveness and variability of the plan, every dish is characterized by its role in the lunch. Hence, dishes are partitioned in 3 **use groups:** every lunch is composed of a starter, a main dish and a dessert chosen among 20 starters, 20 main dishes, and seven desserts, respectively. Hence, 2,800 different lunches are possible.

Nutritional adequacy of the lunch plan is guaranteed by the following constraints:

- The caloric content of every lunch must be in an appropriate interval;
- The total contents of fats, proteins, and carbohydrates over the whole planning period must be in an appropriate interval;
- Fiber, calcium, iron, potassium, magnesium, vitamin A, vitamin C, and vitamin E are considered as micronutrients to be encouraged, so that their content in every lunch is constrained to be greater than an appropriate minimum value;
- Cholesterol and sodium are considered as nutrients to be limited so that their content in every lunch is constrained to be lesser than an appropriate maximum value;
- The total content of saturated fatty acids over the whole planning period is constrained to be lesser than an appropriate maximum value.

The values of the lower and upper bounds of the constraints are retrieved from the Spanish literature (58). All these constraints are linear relationships over the quantities $Q_{d,w}^{p}\left(x\right)$ and *Q*^*p*^(*x*). Most of these constraints must be satisfied exactly (*hard constraints*) while some of them are instead implemented as *soft constraints*, that is the unwanted deviations of $Q_{d,w}^{p}\left(x\right)$ and *Q*^*p*^(*x*) from the lower and upper bounds are minimized by introducing these deviations in the optimization function. To this end some additional continuous variables representing these deviations are introduced. The constraints are then formalized by means of 7 inequalities constraints and 240 equality constraints

The composition of every lunch, that must be composed by a starter, a main dish and a dessert, is guaranteed by means of three constraints on the rates *R*^*g*^(*x*) of the three food groups. Besides the composition of every lunch, there are some other constraints related to attractiveness and variability of the plan. In particular, the starters and the main dishes cannot be repeated more than twice in the planning period and the desserts cannot be repeated more than six times. These constraints are linear and are formalized by means of 47 inequalities on the rates *R*^*i*^(*x*) of every dish *i*.

Finally, the cost and the carbon footprint of the menu are constrained to be as close as possible to given target values. Even in this case, these constraints are implemented as soft constraints over the positive deviations of *Q*^*price*^(*x*) and *Q*^*CF*^(*x*) from the corresponding targets.

The objective function is a linear combination of four terms that are the positive deviations of the carbon footprint and the price of the menu, and the average daily deviations of the energy content and nutrients. Targets for the carbon footprint and the price are obtained considering different percentiles of the 2,800 “repetitive” menus consisting in the same lunch for all the period of the menu itself.

**Figure 2:**
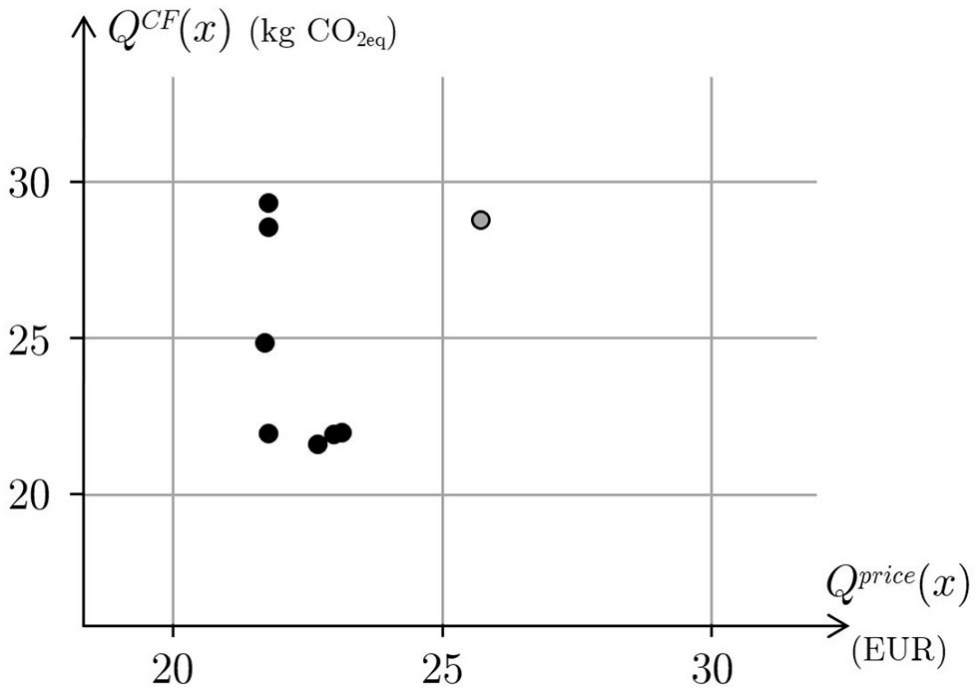
The price and the carbon footprint of several optimal menus obtained for different desired targets of price and carbon footprint (black circles). The gray circle represents the price and the carbon footprint of the average menu.

Then, a first group of seven optimal menus are obtained by pairing the carbon footprint targets as percentiles from the 20th to 90th with price targets as percentiles from the 20th to 90th. The carbon footprint and the price of these menus are plotted in Figure 2 along with the average values of the carbon footprint and the price of the 2,800 repetitive menus.

The figure shows that the price is quite constant for different carbon footprint targets (vertical part of the curve) and the carbon footprint is also quite constant for different price targets (horizontal part of the curve). With respect to the average value of the carbon footprint, the reductions are around 23−24% in the horizontal part of the curve, whereas compared with the average price, the reductions are around 15−16%.

**Figure 3:**
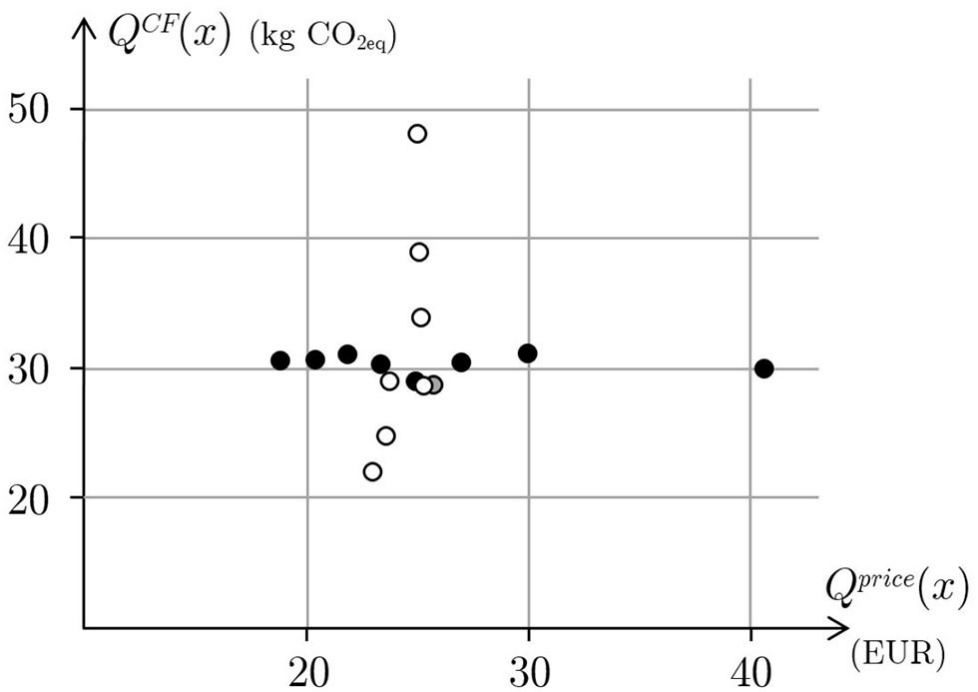
The black circles represent the price and the carbon footprint of several optimal menus obtained for different targets of price while removing price and carbon footprint from the objective function. The white circles represent the price and the carbon footprint of several optimal menus obtained for different targets of carbon footprint while removing price and carbon footprint from the objective function. The gray circle represent the price and the carbon footprint of the average menu.

The influence of the price on the carbon footprint is tested by taking price and footprint deviations out of the objective function and forcing the price to be below the target values. The same thing is done to test the influence of the carbon footprint on the price. The carbon footprint and the price of the menus thus obtained are depicted in Figure 3. The evolution of the carbon footprint according to the different targets for the menu price is an almost horizontal line, around 31 *kg* of *CO*_2*eq*_ and, from the nutritional point of view, lower prices exhibit a similar level of deviation from the nutritional targets to the higher prices. Similarly, the evolution of the price according to the different targets for the carbon footprint is an almost vertical line, around 24 Euros.

It's worth noting that some targets are not achieved for all the optimal menus. This is due to the fact that some nutritional constraints have been modeled as soft constraints. In particular, this is the case of calcium and vitamin E.

### 3.2. Case 2: Another 20-Days School Lunch Plan

The case study (35) consists of finding the optimal lunch plans for school children in Italy for a 20-days (5 days a week for 4 weeks) planning period. Hence, also in this case, *N*_*M*_ = 1, *N*_*D*_ = 5 and *N*_*W*_ = 4. A total set of *N* = 106 dishes is considered and they are retrieved from a guide of health and nutritional practices for the primary school established by the municipality of Rome, Italy (37). This guide provides in fact a set of possible recipes along with the weight of their ingredients and the cooking procedure. Therefore, the number of binary variables is equal to *N* × *N*_*M*_ × *N*_*D*_ × *N*_*W*_ = 2,120. The recipes considered refer to children 6–10 years old, regardless of gender.

The following parameters are associated to each dish:

- 7 **nutritional parameters:** the caloric content together with six among macronutrients and micronutrients (protein, fat, carbohydrate, sugar, fiber and sodium) contents of the dish are calculated from the ingredients using the database of the Italian Council for Agricultural Research and Analysis of the Agricultural Economy (52);
- 2 **environmental impact parameters:** the carbon and water footprints of the dish are calculated from the ingredients using the World Wide Fund for Nature database (54).

Moreover, in order to take into account cultural habits and to guarantee an healthy meal and attractiveness and variability of the plan, every dish is characterized by its role in the lunch and its main ingredient. Hence, dishes are partitioned in the following groups:

- 5 **use groups:** every lunch is composed of a first course, a second course, and a side dish. They are chosen among 33 first courses, 48 second courses, and 23 side dishes, respectively, and served together with bread and fruit. Hence, 36,432 different lunches are possible;
- 12 **food groups:** pasta, tomato pasta, no tomato pasta, rice, meat, fish, eggs, dairy, potatoes, legumes, salads, and vegetables.

Nutritional adequacy of the lunch plan is guaranteed by the following constraints:

- The caloric content of every lunch must be in an appropriate interval;
- The contents of carbohydrates, fiber, and sodium of every lunch must be in an appropriate interval;
- The content of proteins, fats, and sugar of every lunch is constrained to be lesser than an appropriate maximum value.

The values of the lower and upper bounds of the constraints are retrieved from the levels of reference intake of nutrients and energy for the Italian population (10). All these constraints are linear and are formalized by means of 220 inequalities constraints over the quantities $Q_{d,w}^{p}\left(x\right)$.

The composition of every lunch is ensured by a set of 100 equality constraints. Besides the composition constraints, there are some other constraints related to attractiveness and variability of the plan. In particular, lasagna has to be served exactly once in the menu while all the other dishes may be served at most once in a week and twice in the whole plan. Further, variability is ensured by limiting some food categories repetition on weekly scale. For example, meat has to be served at least once in a week but no more than twice. Eggs have to be served exactly once in a week while legumes may not be served at all in a week but can be served up to three times. All these constraints are linear and are formalized by means of 534 inequalities constraints and 31 equality constraints on the rates $R_{w}^{i}\left(x\right)$, *R*^*i*^(*x*), and $R_{w}^{g}\left(x\right)$ of every dish and food group, respectively.

**Figure 4:**
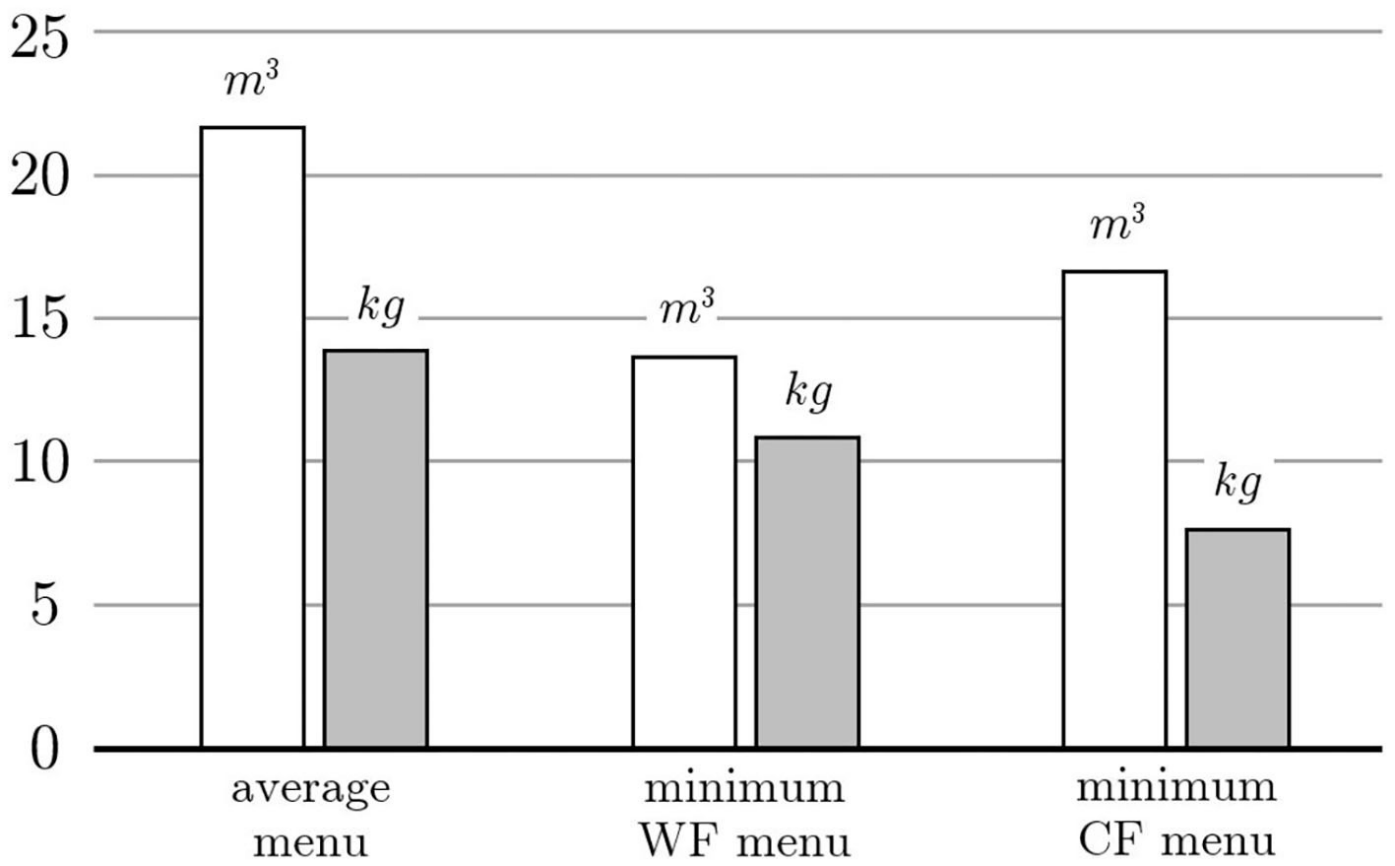
The white and gray bars represent the water and carbon footprint of the average menu or those obtained by minimizing either the water or the carbon footprint.

Two objective functions are considered in order to compare the difference of the menus corresponding to minimizing the carbon or the water footprints, i.e., *Q*^*CF*^(*x*) and *Q*^*WF*^(*x*), respectively. The optimal menu that minimizes the carbon footprint needs 7.77 *kg* of *CO*_2*eq*_ to be served while consuming 16.64 *m*^3^ of water. On the other hand, the optimal menu that minimizes the water footprint needs 13.72 *m*^3^ of water to be served while producing 10.85 *kg* of *CO*_2*eq*_. These values are compared with those of the average menu in Figure 4.

In more detail, the menu with minimum carbon footprint saves more than 40% of the GHG emissions and more than 20% of the water consumed. On the other hand, the menu with minimum water footprint saves more than 35% of the water consumed and more than 20% of the GHG emissions. It is remarkable that both footprints decrease with a significant reduction of the one that is optimized. Moreover, the two menus are equivalent from a nutritional point of view since the average values of energy and nutrients contents are practically the same. Finally, both menus are varied since they make use of the largest possible number of recipes. This is the case because the values of energy and nutrients span over almost all the allowable ranges.

### 3.3. Case 3: A 14-Days Full–Board Nursing Homes Meal Plan

The case study (36) consists of finding the optimal menus for nursing homes in Italy for a 14-days (7 days a week for 2 weeks) planning period. In this case, *N*_*M*_ = 3, *N*_*D*_ = 7, and *N*_*W*_ = 2. A total set of *N* = 143 possible dishes is retrieved from a national sample of Italian nursing home menus by extracting different recipes along with the weight of their ingredients. Therefore, the number of binary variables is equal to *N* × *N*_*M*_ × *N*_*D*_ × *N*_*W*_ = 6,006. The menu is designed for an energy daily intake of 1,800 ± 10% kcal, which corresponds to the reference intake for females 60–74 years old with a low physical activity level.

The following parameters are associated to each dish:

- 5 **nutritional parameters:** the caloric content together with four among macronutrients and micronutrients (protein, fat, carbohydrate, and sugar) contents of the dish are calculated from the ingredients using the database of the French Agency for Food, Environmental and Occupational Health & Safety (53);
- 1 **environmental impact parameter:** the carbon footprint of the dish is calculated from the ingredients using the Carbon Scope Data LCI database and the CleanMetrics food carbon emission calculator (57);
- 1 **economic parameter:** the cost of the dish is determined collecting the prices of its ingredients from a sample of local stores considering the mean value price while ignoring prices on specials.

Moreover, in order to take into account cultural habits and to guarantee an healthy meal and attractiveness and variability of the plan, every dish is characterized by its role in the meal and its main ingredient. Hence, dishes are partitioned in the following groups:

- 8 **use groups:** every breakfast is composed of one recipe from the cereals group (cornflakes, biscuits, rusks, …), beverages group (milk, coffee, tea, juice, …) and sweeteners group (sugar, honey, jam, …). These groups are composed of 8, 5, and 4 different elements, respectively. Hence, 160 different breakfasts are possible. On the other hand, lunches and dinners are composed of a first course, a second course, side dish, bread, and fruit. They are chosen among 47 first courses, 55 second courses, 15 side dishes, respectively, and served with bread and fruit among 2 possible different kind of bread and 9 different fruits. Hence, 697,950 different meals are possible;
- 18 **food groups:** pasta, rice, soup, red meat, white meat, processed meat, fish, eggs, cheese, potatoes, legumes, zucchini, spinaches, eggplants, mushrooms, artichokes, tomatoes, and tuna fish.

Nutritional adequacy of the lunch plan is guaranteed by the following constraints:

- Breakfast is constrained to provide more than 10% of daily energy content;
- The daily caloric content must be in an appropriate interval;
- The daily contents of proteins, carbohydrates and fats must be in an appropriate interval;
- The daily content of sugar is constrained to be lesser than an appropriate maximum value.

The values of the lower and upper bounds of the constraints are retrieved from the levels of reference intake of nutrients and energy for the Italian population (10). All these constraints are linear and are formalized by means of 140 inequalities constraints over the quantities $Q_{d,w}^{p}\left(x\right)$.

The composition of every meal is ensured by a set of 28 inequalities constraints and 252 equality constraints. Besides the composition constraints, there are some other constraints related to attractiveness and variability of the plan. In particular, daily, weekly, and total rates of dishes are limited. For example, first and second courses cannot be served more than once in the entire menu while side dishes can be provided at most once a day, twice a week and three times in the whole plan. Furthermore, also daily, weekly, and total rates of dishes in specific food groups are limited. For example, recipes containing fish can be served at most once a day, and between two and three times a week. Moreover, they must be served at least five times in the whole menu. On the contrary, a limited consumption of red meat is obtained imposing that it can be served exactly once in a week. All these constraints are linear and are formalized by means of 842 inequalities constraints on the rates $R_{d,w}^{i}\left(x\right)$, $R_{w}^{i}\left(x\right)$, *R*^*i*^(*x*) and $R_{d,w}^{g}\left(x\right)$, $R_{w}^{g}\left(x\right)$, *R*^*g*^(*x*) of every dish and food group, respectively.

**Figure 5:**
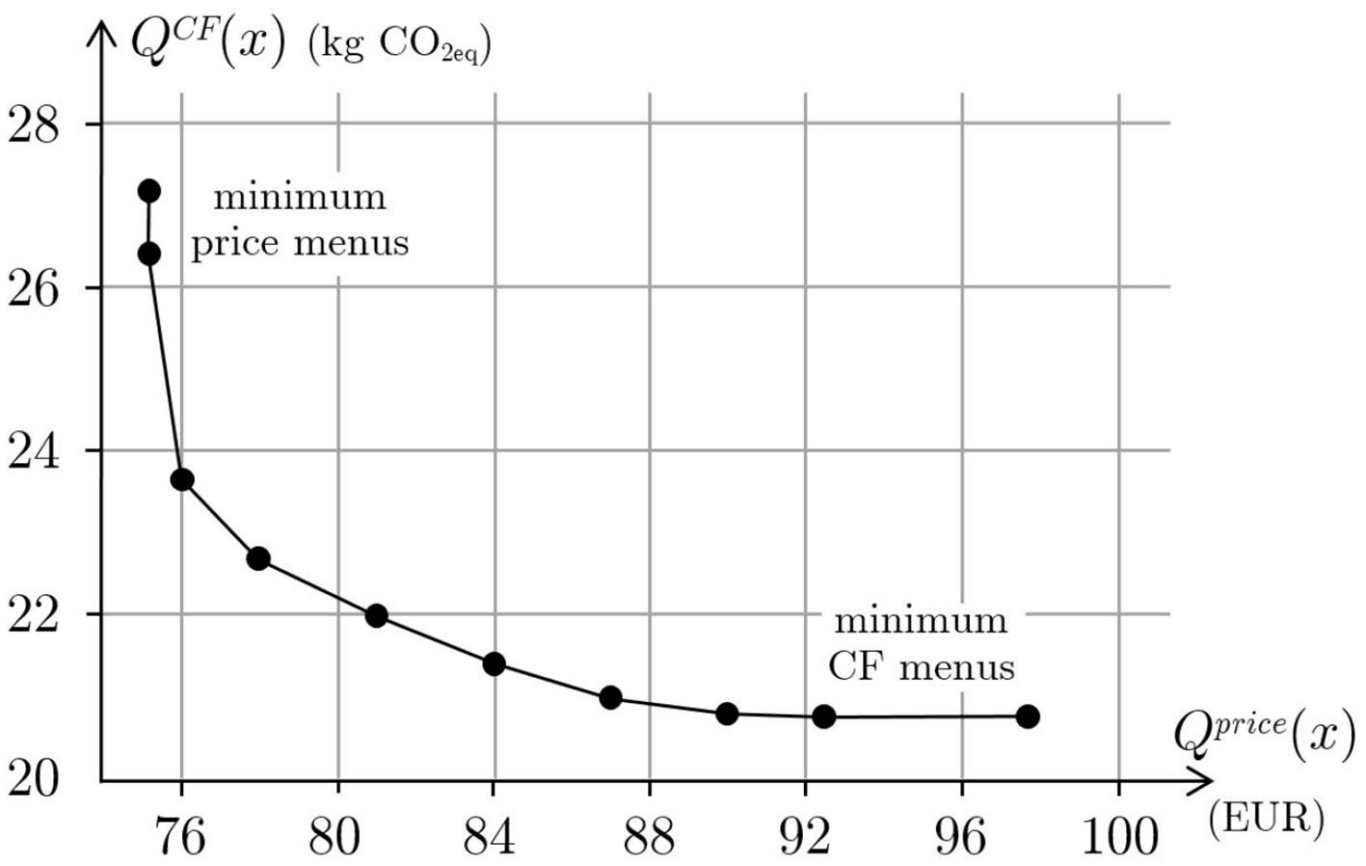
Relation between the price and the carbon footprint of optimal menus.

The problem considered is a multi-objective optimization problem with two goals. They are the cost and the carbon footprint of the menu, i.e., *Q*^*price*^(*x*) and *Q*^*CF*^(*x*), respectively. First of all, the minimum carbon footprint and the minimum price of any feasible menu is determined. These minima are obtained by considering two single objective optimization problems and amount to $Q_{min}^{CF}=20.7$ *kg* of *CO*_2*eq*_ per person and $Q_{min}^{price}=75.15$ Euros. There are several menus with minimal price and their carbon footprint is within 26.4 and 27.2 *kg*. Any of such a menu corresponds to a point on the vertical segment of the curve in Figure 5. Similarly, there are several menus with minimal carbon footprint and their price is within 92.43 and 97.64 Euros. Any of such a menu corresponds to a point on the horizontal segment of the curve in Figure 5. Therefore, there does not exist a menu that simultaneously optimizes price and carbon footprint and this means that the two objective functions are conflicting: environmental friendly menus are more expensive and affordable menus have a higher environmental impact. The trade off between these two goals can be evaluated by means of the Pareto optimal set. This set is shown in Figure 5 and has been computed scalarizing the problem with the ϵ-constrained method, i.e., by solving a sequence of single objective optimization problems, under the same sustainability constraints, with the carbon footprint as objective function and the price constrained to be below an upper bound ϵ varying between 75.15 and 92.43 Euros.

**Figure 6:**
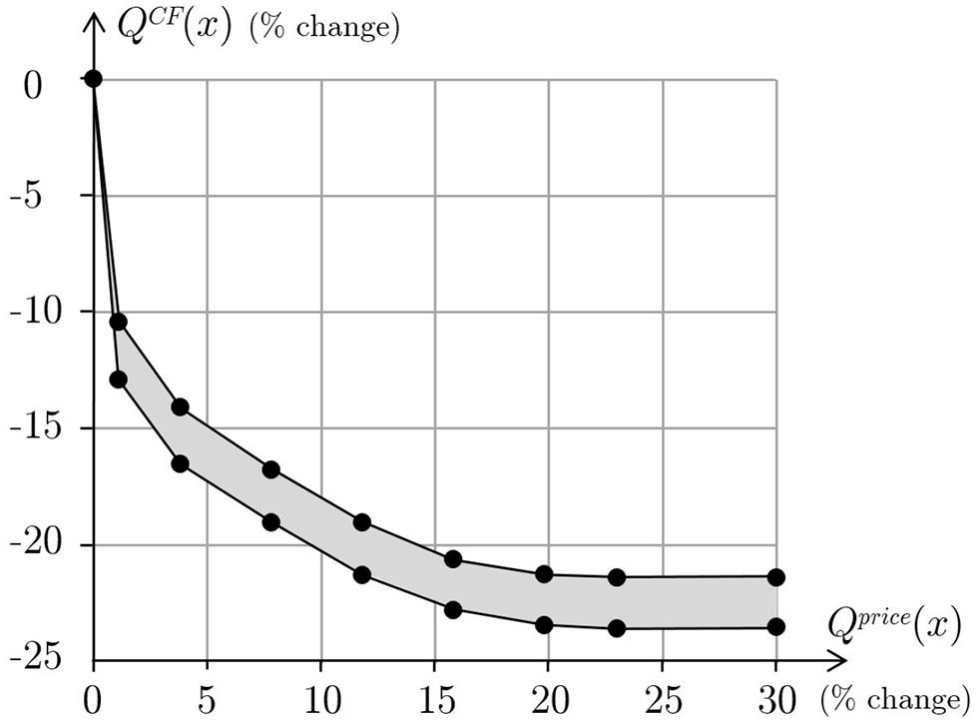
Carbon footprint percentage change as a function of the percentage change of price, with respect to carbon footprint and price of the menus with minimum price.

All the Pareto optimal menus are equally good from a nutritional, acceptability and health point of view. Moreover, the study shows that all these menus are mainly equivalent also with respect to the distribution of energy, proteins, fats and carbohydrates daily contents over the 14 days of the menu. In addition, menus with a small difference in price are substantially the same, that is only few recipes are substituted with others. As Figure 5 clearly shows, the relation between carbon footprint and price is quite smooth. Moreover, the environmental impact of the menu is in a kind of inverse proportion to the menu price. If the quality of a menu is evaluated only in terms of nutritional and health characteristics and acceptability, then any feasible menu obtained with the proposed model, including any Pareto optimal menu, is equally good. Hence, if price priority is chosen, then menus corresponding to points in the left hand side of the curve will be considered. In particular, the best choice will correspond to a menu at minimum price corresponding to a point on the vertical segment of the curve. This is the usual choice of nursing home management that wants to reduce costs while ensuring residents to receive a varied and healthy diet that meets their nutritional needs. As discussed in the introduction, more sustainable choices should instead balance economic and environmental issues. To do this, it is sufficient to note that the curve is very steep on the left, that is for high values of carbon footprint, so that a small increase of the menu price will determine a great decrease of the GHG emissions. Figure 6 shows the carbon footprint percentage change as a function of the percentage change of price where the changes are computed with respect to the values of the menus with minimum price.

For instance, the menu corresponding to an increment in price of about 1%, determines a reduction of more than 10% on GHG emissions. Similarly, the menu corresponding to a reduction of about 15% of GHG emissions costs just 4% more than the minimum price.

## 4. Discussion

The three case studies above illustrated make use of the advanced optimization model presented in this paper with increasing order of complexity. Table 1 summarizes some indicators of the structure of the models used to design these meal plans.

**Table 1:** Indicators of the structure of the models used to design the meal plans in case 1, case 2, and case 3.

	**Case 1**	**Case 2**	**Case 3**
**PLAN CHARACTERISTICS**			
Meals a day	1	1	3
Days per week	5	5	7
Weeks	4	4	2
Dishes	47	106	143
Different possible meals	2,800	36,432	698,110
Different possible plans	≈10^69^	≈10^91^	≈10^278^
**Binary variables**	940	2,120	6,006
**Continuous variables**	484	0	0
**PARAMETERS AND GROUPS**			
Nutritional parameters	14	7	5
Environmental impact parameters	1	2	1
Economic parameter	1	0	1
Use groups	3	5	8
Food groups	0	12	18
**Total parameters and groups**	19	26	33
**CONSTRAINTS**			
Nutritional constraints	247	220	140
Env. impact constraints	1	0	0
Economic constraints	1	0	0
Meal composition constraints	3	100	280
Variability constraints	47	565	842
**Total constraints**	299	885	1,262
Equality constraints	242	131	252
Inequality constraints	57	754	1,010

Case 1 and case 2 deal with the same scenario and have a similar meal structure. Nevertheless, in case 2, more recipes are made available so that the number of possible lunches increases of one order of magnitude. Case 3 is definitely a different problem since all the meals of every day are considered and the number of recipes is even larger than case 2, thus resulting in an increase of the number of possible menus of one further order of magnitude. For the same reason, the number of binary variables of the model doubles from case 1 to case 2, and triplicates from case 2 to case 3. This already indicates an increasing complexity of the studies, which is also determined by a larger number of parameters and groups from one case to the other. As the table makes clear, the parameters used in case 1 are mainly nutritional and no food groups are considered. This is due to the fact that acceptability in case 1 is very basic and it is pursued only through the definition of three use groups. Case 2 and case 3, besides nutritional issues, are instead more oriented in ensuring attractiveness and variety of the menu, and this is achieved by defining a significant number of food groups. The increasing number of binary variables and that of parameters and groups results in an increasing total number of constraints in the three studies. As expected, case 1 has mainly nutritional constraints, while the other two cases have a predominant number of constraints modeling the cultural acceptability of the menu and its attractiveness.

Beside complexity, the main difference in these three cases is the way some nutritional recommendations are modeled. In particular, in case 1, some of them are not considered as mandatory (hard constraints) but rather as targets to be achieved as close as possible (soft constraints). This means that it is possible not to fulfill the recommendations while though minimizing deviations from them. To do this, additional continuous variables representing the deviations must be introduced and, at the same time, these deviations must be considered in the objective function. Therefore, a mixed optimization problem, that is a problem with both discrete and continuous variables, is obtained. To model positive and negative deviations from the bounds defining the recommendations, 484 continuous variables are introduced in case 1. Consequently, the objective function is composed by terms regarding the original goals of the problem (menu price and carbon footprint) and terms representing the deviations. Hence, the solution does not minimize the original term alone but yield a possible trade off with the deviations from the nutritional recommendations. As a matter of fact, the optimal solutions considered in case 1 do not fulfill some nutritional constraints in an hard way, see calcium and vitamin E. Anyway, from a modeling point a view, case 1 is the first effort to move from the classical LP model to the new paradigm presented in this paper. It results to be slightly involved just because it encompasses most of the features of this new methodology but still retains some features of the LP design.

The new paradigm is instead completely explored in case 2 and case 3. In these cases the need to take into consideration acceptability issues (25) has been fully received. As a matter of fact, a significant number of use and food groups has been introduced to comply with the structure of the meals (cultural habits) and to provide an attractive and varied menu. Moreover, only hard constraints are considered so that no additional continuous variables are needed. Therefore, the problem remains BLP and, in the optimal solution, all the recommendations are strictly satisfied.

Case 2 and case 3 differ for the choice of the objective function. In fact, each one of the two problems considered in case 2 has a single objective function while that in case 3 results in a multi-objective optimization problem.

The study presented in case 3 takes into account acceptability in a very extended way and it shows how to pursue optimality when conflicting goals are considered. The Pareto optimal set represents in fact the optimal tradeoffs between the competing goals and moving over this set allows to chose which blend is preferred. This new concept, along with modeling the meal plan design as an optimal allocation of resources, is a well-established and validated practice in engineering problems but, to the best of authors knowledge, it is completely innovative in the nutrition field.

## 5. Conclusions

Food consumption patterns dictate the shape of the global food production system. In few next decades, the world population food demand will be almost doubled. To tackle the challenge of achieving and sustaining an adequate food production level whilst guaranteeing environmental integrity and public health, a substantial change in the food consumption patterns must be proposed, toward more sustainable diets. The concept of sustainability integrates different dimensions: nutrition, health, environmental impact, socio-economic impact, and cultural acceptability. The latter in particular is a critical issue in encouraging people in shifting their eating habits toward a new pattern. Mathematical optimization models have proved to be successful in designing optimal food plans: these are selected combinations of foods that generally minimize the environmental impact of the diet, while satisfying proper nutritional recommendations. The food plan consists of the level of consumption of selected foods, but no practical meal plans – that is schedules of recipes – are provided. Moreover, acceptability can only be dealt with in an uneasy indirect way, making diets attractiveness quite questionable. In this paper a new modeling paradigm to design sustainable diets has been presented. Realistic meal plans are obtained providing the sequence and composition of daily meals over a given period of time. Each meal is composed using dishes from a given set of culturally acceptable recipes for the target population, and the meals structure also obeys to local eating habits. The optimal menu can be designed by defining appropriate criteria for dishes selection according to the competing goals of a sustainable diet while ensuring meals attractiveness and variability. From a mathematical point of view, a 0–1 binary integer linear programming optimization problem is obtained where acceptability can be explicitly taken into account in a very natural way, that is bounding the daily, weekly, or total repetitions of single dishes, dishes with similar ingredients and dishes in the same food groups; pairing of dishes in every meal can be taken into account as well. Indeed, the proposed methodology considers a menu as an optimal allocation of resources (dishes) over the period of the diet. The optimization model is scalable, i.e., it is capable to cope with an increased data size. In other words, one can easily consider more recipes, parameters, food and use groups as well as different constraints without affecting the structure of the model. Increasing the size of the model makes the corresponding BLP problem more difficult to solve with respect to the LP problems usually considered in nutrition to define food plans (25). This is mainly due to the fact that a food plan consists of the level of consumption of selected food groups that therefore assumes continuous values. Hence, for the corresponding LP problem, the simplex algorithm is a well-established universal algorithm to compute the global optimal solution. On the contrary, the computation of a solution to a BLP problem can be an extremely difficult task. In fact, despite one might expect that restricting the range of the variables to binary values would simplify the computations, just the opposite happens (59). This depends from the binary nature of the variables that does not allow the use of searching methods requiring continuous variation of the variables themselves. The optimization problem can be efficiently solved by available dedicated software tools like LINGO and AMPL. Three meaningful case studies recently appeared in literature have been reviewed, showing the effectiveness of the proposed methodology at different level of complexity of the acceptability constraints. The reported results show a great reduction of the environmental impact of the meal plans while ensuring an adequate nutritional intake, affordable prices and most importantly the plans are varied and culturally acceptable. The sets of dishes considered in the three case studies are recipes usually available in schools or nursing homes. Hence, the results show that sustainability can be improved at no cost since the method just provides a smart schedule of meals without requiring different recipes from the usual ones. As a consequence, the method can well be applied to some other food service areas, such as company service canteens, chain restaurants or other individual establishments. Moreover, more sophisticated constraints related to specific nutritional requirements (diets for diabetes, celiac disease, …) can be considered as well.

## Data Availability Statement

The raw data supporting the conclusions of this article will be made available by the authors, without undue reservation.

## Author Contributions

LB and AD: study conception and design, acquisition, analysis and interpretation of data, and writing article. All authors contributed to the article and approved the submitted version.

## Conflict of Interest

The authors declare that the research was conducted in the absence of any commercial or financial relationships that could be construed as a potential conflict of interest.

## Footnotes

^1^The size of the portion depends in general on age, weight, gender, level of physical activity, …

^2^The recipe of the dish (37) consists of 70 g of dried pasta, 80 g of peeled tomatoes, 5 g of carrots, 5 g of onion, 2 g of celery, 5 g of parmesan, and 4 g of extra-virgin olive oil.

^3^In the case the energy intake is allowed to be in 2,000 ± 10% kcal, then the share of carbohydrates changes to 202.5−357.5 g, the lower bound calculated as the 45% of the lower energy limit and the upper bound as the 65% of the upper energy limit.

^4^A food group is defined as a collection of foods or dishes that share similar nutritional properties or biological classifications. Moreover, in order to guarantee variability of the plan, this definition can be extended to a collection of dishes sharing the same main ingredient.

^5^These methods allow, in general, to compute the set of weak Pareto optimal solutions, that is solutions for which some of the objectives could be improved while keeping others unchanged. This set contains the Pareto optimal set and can be considered as a narrow approximation of it.

^6^In the Italian infant primary and secondary school, i.e., children from 3 to 13 years old, schools with canteen service are in charge to prepare the meals according to a guide of health and nutritional practices established by municipalities. The guide provides requirements to ensure food safety and hygiene as well as proper nutritional intakes. In more detail, the municipality of Rome provides a set of possible recipes along with the weight of their ingredients and the cooking procedure (37).

^7^This database was made in collaboration with the University of Tuscia at Viterbo (Italy), the Second University of Naples (Italy) and Mutti S.p.A., on the basis of the databases and research reports (55, 56).
